# Amygdala downregulation training using fMRI neurofeedback in post-traumatic stress disorder: a randomized, double-blind trial

**DOI:** 10.1038/s41398-023-02467-6

**Published:** 2023-05-25

**Authors:** Zhiying Zhao, Or Duek, Rebecca Seidemann, Charles Gordon, Christopher Walsh, Emma Romaker, William N. Koller, Mark Horvath, Jitendra Awasthi, Yao Wang, Erin O’Brien, Harlan Fichtenholtz, Michelle Hampson, Ilan Harpaz-Rotem

**Affiliations:** 1 Centre for Cognitive and Brain Sciences, University of Macau, Macau SAR, China; 2 Department of Radiology and Biomedical Imaging, Yale University School of Medicine, New Haven, CT USA; 3 Department of Psychiatry, Yale University School of Medicine, New Haven, CT USA; 4 National Center for PTSD, West Haven, CT USA; 5 Department of Psychology, Keene State College, Keene, NH USA; 6 Child Study Center, Yale University School of Medicine, New Haven, CT USA; 7 Department of Biomedical Engineering, Yale University School of Medicine, New Haven, CT USA; 8 Department of Psychology and Wu Tsai Institute, Yale University, New Haven, CT USA

**Keywords:** Psychiatric disorders, Neuroscience

## Abstract

Hyperactivation of amygdala is a neural marker for post-traumatic stress disorder (PTSD) and improvement in control over amygdala activity has been associated with treatment success in PTSD. In this randomized, double-blind clinical trial we evaluated the efficacy of a real-time fMRI neurofeedback intervention designed to train control over amygdala activity following trauma recall. Twenty-five patients with PTSD completed three sessions of neurofeedback training in which they attempted to downregulate the feedback signal after exposure to personalized trauma scripts. For subjects in the active experimental group (*N* = 14), the feedback signal was from a functionally localized region of their amygdala associated with trauma recall. For subjects in the control group (*N* = 11), yoked-sham feedback was provided. Changes in control over the amygdala and PTSD symptoms served as the primary and secondary outcome measurements, respectively. We found significantly greater improvements in control over amygdala activity in the active group than in the control group 30-days following the intervention. Both groups showed improvements in symptom scores, however the symptom reduction in the active group was not significantly greater than in the control group. Our finding of greater improvement in amygdala control suggests potential clinical application of neurofeedback in PTSD treatment. Thus, further development of amygdala neurofeedback training in PTSD treatment, including evaluation in larger samples, is warranted.

## Introduction

Post-traumatic stress disorder (PSTD) is a debilitating disorder with a high lifetime prevalence worldwide [1]. Individuals with PTSD typically experience symptoms of re-experiencing, avoidance, alterations in cognition and mood, and marked alterations in arousal and reactivity.

With its neurobiology extensively studied in rodents and humans, although far from perfectly understood, PTSD fear responses are considered to be one of the better understood neural mechanisms underlying the disorder. Neuroimaging studies in PTSD have highlighted neuroplastic changes in brain structures including the prefrontal cortex (PFC), hippocampus, thalamus, and amygdala [2, 3]. Given its role in fear conditioning and processing of negative emotions, the amygdala is an area of particular interest for PTSD. Indeed, hyperactivation in the amygdala complex is a prominent brain marker of the disorder [4, 5]. Clinical studies using psychotherapy have reported symptom reductions associated with decreases in amygdala activity following treatment [6]. Furthermore, successful MDMA (3,4-Methylenedioxymethamphetamine) treatments appear to facilitate healthy trauma recall by decreasing amygdala activity [7] and preliminary deep brain stimulation (DBS) studies targeting amygdala activity have reported promising findings of this neuromodulation technique in reducing PTSD symptoms [8]. These findings together suggested that suppressing fearful amygdala response in PTSD is a promising avenue for treatment.

In recent decades, there has been encouraging progress in developing real-time fMRI neurofeedback training as an intervention for treating mental illnesses including PTSD. A number of these studies have focused on normalizing amygdala activity with the goal of enhancing emotion regulation [9–12]. As PTSD symptoms stem from individual traumatic experiences [1], we aimed to train participants to reduce amygdala activity following personal trauma recall using fMRI neurofeedback. Our pilot study in treatment-refractory PTSD suggested this approach could lead to meaningful symptom reduction [13]. In this double-blind, randomized clinical trial we examined whether our amygdala neurofeedback training protocol could help patients with PTSD to control their amygdala activity following trauma recall and thereby improve symptoms.

## Materials and methods

This study was pre-registered on ClinicalTrials.gov (NCT03574974). The study protocol was reviewed and approved by Yale IRB (approval number #2000022668).

The brain imaging sessions were carried out on a 3-Tesla MRI system (imaging details in Supplementary Information (SI)). Online fMRI data preprocessing and neurofeedback were performed using the real-time module in BioImage Suite 3.0. We used MATLAB (The MathWorks Inc.; Natick, Massachusetts) for stimuli (including feedback signal) presentation. Statistical tests on the extracted imaging and clinical measurements were performed with JASP (https://jasp-stats.org/).

### Participants

The target sample size for the current study was twenty-four participants in total based on the funding support we received. Individuals with PTSD symptoms were recruited via online advertisement and community outreach. After passing a short phone screening, potential participants were assessed by a clinical psychologist using the Structural Clinical Interview for DSM-IV (SCID-IV) and the Clinician-Administered PTSD Scale for DSM-5 (CAPS-5). Those who were diagnosed with chronic PTSD and met all other inclusion criteria (see SI for full details and CONSORT flowchart (Fig. S1)) were invited to participate in the study. We obtained written informed consent from all participants.

### Stimuli preparation

After screening for symptoms, eligible candidates met with a clinician who collected information regarding their personal traumatic experiences and were prompted to report physiological responses associated with the traumas. Each traumatic experience was rated for its intensity by the participant. The personal trauma narratives were subsequently transcribed following the methods described by [14, 15]. Two versions of each traumatic event were created with slightly different wording and highlighting different aspects of the physiological responses reported. These scripts were recorded into two one-minute audio clips by a male and a female native English speaker. In addition, noises associated with the trauma were concatenated into a temporal sequence that mirrored the sounds heard leading up to and during the traumatic event. In this way, for each traumatic experience described, a number of audio clips were created that evoked memories of the event (some scripts and some noise clips). These audio clips were played in the subsequent brain imaging tasks through headphones to provoke amygdala responses.

### Imaging screening and ROI identification

For most participants, the localizer session took place approximately 3 weeks after the initial CAPS interview. Due to the COVID-19 pandemic and other scheduling difficulties, the localizer scan could not be completed in this time frame by some participants. For these participants, CAPS score was re-collected to ensure the recency of the measurement. During the functional localizer session, the participants were instructed to listen to personalized trauma scripts and refrain from controlling their anxiety (see SI for details of the localizer task). Data from the three localizer runs were used to identify the most active amygdala area during trauma exposure under a standard analytic procedure that has been previously described [13, 16]. In brief, generalized linear model (GLM) analysis was performed to identify activation during the provocation periods in the functional data collected from the localizer task (details of the GLM analyses in SI). The thresholded t-map from the GLM analysis was smoothed and then manually edited to exclude non-amygdala areas. Voxels with signal dropout were also excluded using a fixed amplitude threshold. The thirty most active voxels (exceeding a *t* = 1 threshold with each cluster including 4 or more voxels) within the amygdala region were identified from the edited map using an in-house script. Individuals that failed to activate the amygdala sufficiently were not randomized into the trial. This was important given that insufficient amygdala response after trauma recall can signal a potential tendency for dissociation which we did not want to reinforce by including these participants in the trial. Individual regions of interest (ROI) determined from the localizer data were subsequently used for providing neurofeedback to the active group as well as for calculating participants’ improvement in self-regulation and seed-based resting-state functional connectivity maps in the offline analyses.

### Neurofeedback training

**Fig. 1:**
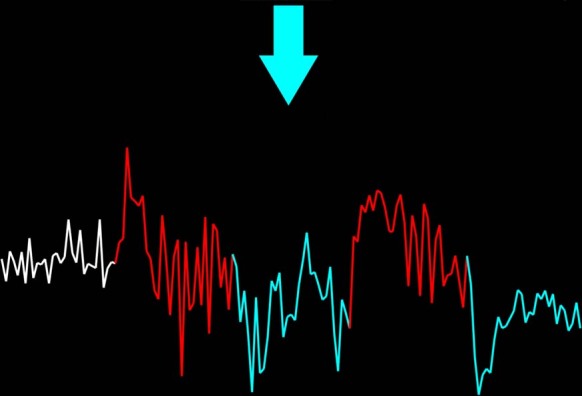
Example of a neurofeedback run. Participants were instructed to rest at the beginning of the run (white period, 1 min) and allow their amygdala activity to increase by listening to the trauma scripts (red period, 1 min each block) and then downregulate the activity during the blue period (1 min each).

Eligible participants were assigned to receive either real neurofeedback (active group) or a yoked-sham form of feedback in which they were matched to a real feedback subject and shown exactly the same time courses (control group). Group assignment was based on a pseudo-random sequence generated by computer program prior to study initiation. The timeline for both groups was similar to our feasibility study [13] consisting of three training sessions with 6 runs per session. Prior to the first session, a clinical psychologist met with the participants and provided personalized mental strategies they could use during the training (details of the strategy development procedure in SI). During the 5-minute training runs, participants were instructed to downregulate the feedback signal after listening to personal trauma scripts (Fig. 1), and that employing the strategies for this was optional. The active group received feedback on amygdala activity in their individualized ROIs while the control group received the recorded feedback from a matched participant from the active group. Participants were encouraged to explore different mental regulation strategies in order to discover the most effective ones. The methods for real-time preprocessing and feedback calculation have been extensively described previously [13, 16].

### Post-intervention debriefing

After completing the study (and before being unblinded), participants were asked whether they thought they received real or control intervention during the neurofeedback sessions and how confident they were with the guess (on a scale of 1 to 10). Debriefing data from these two items were converted to an intervention belief variable by multiplying the guessed group (1 for real and −1 for sham) by level of confidence (1 to 10). This variable was included as a covariate in the follow-up analyses of covariance (ANCOVAs) on the outcome measures.

### Primary and secondary outcome measurements

**Fig. 2:**
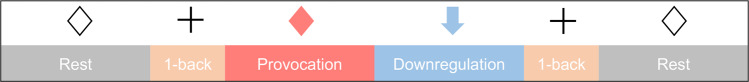
The layout of a control task run. Participants were instructed to allow themselves to experience the anxiety induced by the scripts during the symptom provocation period (cued by a red diamond sign on the screen). Following provocation, they used mental strategies acquired from the strategy development session to decrease the amygdala activity (downregulation period cued by the blue arrow).

Before and after the neurofeedback sessions, participants completed tasks to assess their changes in control over amygdala activity at the following timepoints: pre-NF, post-NF, and 30 days post-NF. Participants completed 4 amygdala control task runs during each of these individual imaging sessions. Each control task run lasted 5 minutes. In these runs, there was a provocation period during which participants heard a minute-long audio clip designed to induce recall of a specific traumatic event. This was followed by a downregulation block when they were cued by a blue down-arrow to decrease activity in their amygdala. These two blocks were embedded in 1-back task blocks (30 s per block) that helped to distract subjects and to prevent anxiety associated with the trauma from affecting the resting periods at the beginning and end of the run (see Fig. 2 which depicts the block timing in these runs). The first two control task runs of each session exposed participants to traumatic events that they were not trained to downregulate during neurofeedback sessions (untrained traumas) whereas the last two control task runs in each session had traumas that they were trained on during neurofeedback (trained traumas). Participants’ ability to downregulate was measured by their amygdala activation level during the corresponding condition as computed in GLM analyses (beta-estimates averaged over the 30 voxels in the ROI). The change in this ability during the untrained trauma control task runs served as the primary outcome measurement of the trial (details of preprocessing and GLM analysis in SI).

CAPS-5 data were collected during screening, 30 days post-NF and 60 days post-NF by clinical psychologists who were blind to the group allocation of the participants. Change over time in CAPS-5 score was the secondary outcome measurement. The last timepoint provides an opportunity to sample slowly developing improvements in symptoms as we have previously discovered in neurofeedback studies that clinical benefits can be slow to unfold following training [17]. PTSD Checklist for DSM-5 (PCL-5) was collected during each study visit to allow a more refined time by group analysis. For these two pre-registered measurements above, we used independent sample *t*-tests to compare their changes since baseline between the two groups. Prior to the *t*-tests, Shapiro-Wilk test was employed to ensure none of these data significantly deviated from a normal distribution as signaled by *p* < 0.05 in this test. For the data that showed significant group differences, their equality of variance was assessed by Levene’s test.

Our secondary outcome measurements also included changes in amygdala resting-state functional connectivity (RSFC). This data was collected pre-NF, post-NF, and 30 days post-NF preceding the control task runs. For each timepoint, two back-to-back five-minute resting-state runs were collected in which the participants were presented with a fixation on the screen and instructed to rest and keep their eyes open. The resting-state data were preprocessed in BioImage Suite in a similar pipeline as the GLM data. Amygdala connectivity was computed using individual ROIs defined from the localizer data. A mixed-design model testing for main effects of group and time, and group-by-time interaction, was computed with MRM toolbox [18].

## Results

Adverse events were monitored at all visits and no adverse events related to the intervention were identified. Among the 27 randomized participants, only two participants dropped out, and these were for reasons that were unrelated to the intervention. Twenty-five participants (14 in the active group and 11 in the control group) completed the training. CAPS-5 data is available in all 25 participants for all three timepoints (screening, 30 and 60 days after neurofeedback). The baseline amygdala control data from one participant in the control group was excluded for excessive head movement during the scan, and five participants (4 in the active and 1 in the control group) failed to complete the 30-day follow-up imaging assessment, see CONSORT flow diagram (Fig. S1) for more details. The baseline characteristics of the completers are reported in Table S1. We tested all the demographic and baseline clinical variables for group effects (using pooled *t* tests for continuous data and Fisher’s exact tests for the binary variables) and there were no significant effects of group.

### Debriefing Results

A higher proportion of subject in the control group (relative to the experimental group) did guess they received the control intervention (7/11 versus 4/14). To explore whether this could be driving group differences, we performed follow-up analyses of our outcome variables controlling for their intervention belief (computed as described in the Post-intervention debriefing section of the Methods). As described in more detail below, the inclusion of this variable in the model did not affect our results.

### Primary outcome measure – change in control over amygdala

**Fig. 3:**
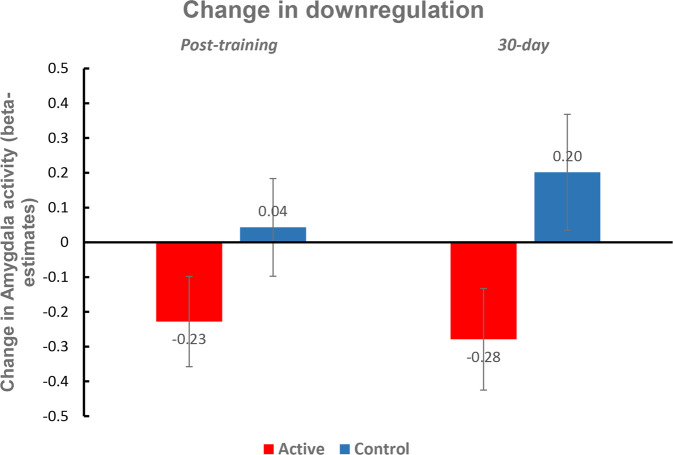
Change in amygdala downregulation ability at the post-training and 30-day time points relative to baseline. Amygdala downregulation ability was assessed for each time point based on amygdala activation during the downregulate blocks of the control task runs involving the traumas that the participants were not trained on during neurofeedback. Error bars represent standard error of the mean.

Changes in amygdala activity during the downregulation condition of the control task runs (involving the untrained trauma) were compared between the two groups with independent sample t-tests. Change from baseline was separately tested for the two post-NF timepoints (post-NF and 30-day follow-up). The group difference at the post-NF time point had a medium effect size in the hypothesized direction (Cohen’s *d* = 0.58) that did not reach significance (*t*_22_ = 1.416, *p* = 0.171, two-tailed). At the 30-day follow-up, however, the group difference had a large effect size (Cohen’s *d* = 0.986) and was significant (*t*_17_ = 2.146, *p* = 0.047, two-tailed). In summary, by the 30-day follow-up time point, the active group had developed a greater ability to reduce amygdala activity (Mean = −0.279, SE = 0.146) during the downregulation block compared with the control group (Mean = 0.202, SE = 0.167; *p* = 0.391 in variance equality test) (Fig. 3). This difference was still significant when including their intervention belief (level of confidence in the treatment received) as a covariable in the comparison (ANCOVA post hoc *t* = 2.173, *p*_tukey_ = 0.045, Cohen’s *d* = 0.999). Note that this group difference was driven not only by an increased ability to reduce amygdala activity in the experimental group but also by a decreased ability to reduce amygdala activity in the control group. One possible explanation for the latter is that motivation on the control task may decline from the start of the study to the end, resulting in performance decrements unless learning counteracts that effect.

### Secondary outcome measure – change in CAPS-5 score

Symptoms (as measured with the CAPS-5) improved significantly for both groups over the course of the study (*t*_24_ = 5.060, *p* < 0.001, Cohen’s *d* = 1.012, paired t-test comparing 60-day follow-up with baseline). Two-sample *t*-tests did not indicate a significant difference in symptom improvements between groups at either 30-day (active group Mean = -8.786, SE = 2.604, control group Mean = −10.545, SE = 4.053; *p* = 0.708, Cohen’s *d* = 0.153) or 60-day follow-up (active group Mean = −13.286, SE = 2.655, control group Mean = −9.364, SE = 3.994; *p* = 0.406, Cohen’s *d* = 0.341), and ANCOVA with the intervention belief as a covariable showed same results (*p* = 0.549 at 30-day follow-up and *p* = 0.163 at 60-day follow-up).

**Fig. 4:**
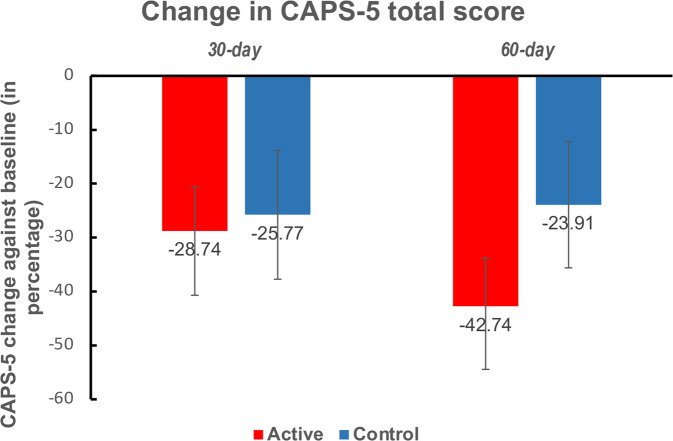
Percent symptom change since baseline, as assessed with CAPS-5. The active group had more pronounced symptom improvements than the control group, but there was no significant group difference at either timepoint. Error bars represent standard error of the mean.

The control group had higher levels of baseline symptoms than the active group, although this difference did not reach significance (*p* = 0.095 comparing the CAPS scores between groups with two-sample t-test, see Table S1 for detailed demographic information and clinical characteristics at baseline). To account for individual differences at baseline, improvements in symptoms are shown in Fig. 4 as percent changes from baseline at the two post-NF time points.

### Secondary outcome measure – change in amygdala RSFC

Mixed-model ANOVA comparing changes in amygdala RSFC between treatment groups across the three assessment timepoints (baseline, post-NF and 30-day follow-up; *n* = 20 as the first five participants did not complete resting-state scans due to a miscommunication) at a whole-brain level did not find significant amygdala connectivity change after correcting for family-wise errors.

### Exploratory analyses

A similar pattern in symptom change was observed in the PCL-5 data as was seen in the CAPS data (*p* = 0.827, Cohen’s *d* = 0.093 and *p* = 0.476, Cohen’s *d* = 0.305 at 30-day and 60-day follow-ups respectively, see details in Change in PCL-5 score in SI).

We did not find any correlation between the reduction in CAPS score and improvement in amygdala control regardless of timepoints examined (smallest *p* = 0.441 using two-tailed Pearson correlation).

## Discussion

In the current study, we demonstrated the effect of amygdala neurofeedback training in PTSD for improving control over amygdala activity following trauma recall.

Our finding of better amygdala control after training converges with data from previous fMRI neurofeedback studies targeting the same region. In one study, PTSD patients were successfully trained to lower bilateral amygdala activation during exposure to personal trauma-related words [19]. Other neurofeedback studies that trained patients with emotion regulation deficits to upregulate their amygdala activity while recalling positive autobiographic memories have also demonstrated improvements in both amygdala activity and connectivity patterns [20–22]. Considering the role of amygdala in trauma recall and stress response [23], these results together suggest the effectiveness of amygdala neurofeedback training for modulating brain circuitry of relevance to PTSD symptoms. It’s worth noting that similar to a previous amygdala-NF study in PTSD [20], our control group showed a trend for decreased amygdala control as indicated by higher activity at 30-day follow-up. This may be attributed to the decline of motivation in exerting self-regulation in this group whereas this effect was counteracted by the neurofeedback-promoted learning in the active group.

Reductions in PTSD symptoms were seen in both groups in this trial. The active group had greater symptom improvements than the control group, but this group difference was not significant. The lack of group difference in clinical change may be due to our relatively small sample size and to the robust clinical improvements associated with exposure therapy alone - both groups were exposed to personalized trauma scripts over the course of the study which is potentially therapeutic for the patients [24]. It is also important to note that the Covid-19 pandemic began midway through this study and may have affected clinical trajectories of the participants. For example, the inhibited social interaction outside of the study may have reduced both avoidance behaviors and triggers and thereby provided therapeutic benefit to patients. Or conversely, as covid restrictions began to lift, engaging in the study and interacting with study staff may have had therapeutic benefit for those who felt isolated prior to study participation. In short, it’s difficult to know how exactly the pandemic impacted these data, but it may have played a role. In any case, a larger study is needed to determine if the neurofeedback provides clinical benefit above and beyond the nonspecific effects of the intervention.

Both our clinical and brain imaging outcome measures bear an intriguing pattern of continuing improvement at later assessment timepoints. This observation is consistent with the clinical data from our other neurofeedback studies that used similar training protocols, one in obsessive-compulsive disorder and one in Tourette syndrome [17]. Furthermore, it suggests the intervention has the potential to induce long-term clinical impacts with minimal need for refresher training sessions: a promising feature given that PTSD frequently has a long-term course [25].

In addition, the clinical promise of amygdala neurofeedback training for PTSD is amplified by ongoing work in the Hendler lab: they have developed an EEG technique for targeting amygdala activity, referred to as EEG Fingerprinting of amygdala activity [26]. The translation of our protocol to EEG in a similar manner could be the focus of future work and could provide a more easily disseminated intervention using the cheaper and more accessible modality of EEG. Indeed, EEG neurofeedback targeting amygdala activity in the context of trauma narratives showed promise in a recently published study [27]. Our protocol differs from the Fruchtman-Steinbok et al. protocol [27] not only in the imaging modality used (fMRI), but also the number of training sessions (3 sessions versus 15), the functional specificity of the region of the amygdala trained (our study targets the peak 30 voxels rather than the full amygdala), and comparison with a blind control group. However, the results converge in suggesting potential clinical promise for neurofeedback protocols that train PTSD patients to downregulate amygdala activity in the context of trauma recall.

Of course, amygdala activity training is only one of many possible approaches for treating PTSD with neurofeedback. In addition to dysregulation of this limbic area, the prefrontal cortex is also implicated in generating symptoms. In rodents, the basal lateral area of amygdala was known to bidirectionally project to the medial prefrontal cortex, a pathway that is central to fear learning and extinction [28] and is weakened in PTSD patients’ brains [29]. Neurofeedback studies targeting the prefrontal cortex as well as anterior cingulate cortex have reported reductions in some symptom clusters [30] and mood improvements [31]. Furthermore, some real-time fMRI neurofeedback studies have shown the effects of prefrontal-amygdala connectivity training in enhancing emotion regulation capacities in healthy participants [32–34]. Thus, there are a number of promising alternative avenues for using neurofeedback therapeutically in PTSD.

In summary, our three sessions of neurofeedback training induced improvements in control over amygdala activity in PTSD patients, and improvements in amygdala control in the active group significantly exceeded those in the control group. However, the clinical data did not demonstrate significantly greater symptom reduction in the active group compared to the control group, likely due to the limited sample size and strong clinical response in the control group in this study. The trajectory of changes seen in both the brain and clinical data suggest neuroplastic changes may develop after the training and highlight the potential for long-term impacts of this intervention.

## Supplementary information

Supplementary Materials

## Data Availability

The extracted amygdala activation values along with the CAPS-5 data are available for download in the NIMH Data Archive (Collection 3042, 10.15154/1527951).
