# Supplementary material for: Amygdala downregulation training using fMRI neurofeedback in post-traumatic stress disorder: a randomized, double-blind trial
Source: Transl Psychiatry. 2023 May 25;13:177. doi: 10.1038/s41398-023-02467-6 (PMC10209552; doi:10.1038/s41398-023-02467-6)
Supplement: Supplementary file 1 — Supplementary Materials [file 41398_2023_2467_MOESM1_ESM.docx]

# Supplementary Materials

**Recruitment**

**Inclusion criteria**

- Ages 18 and up
- Diagnosis of chronic PTSD, as established by the Clinician-Administered PTSD Scale for Diagnostic and Statistical Manual of Mental Disorders, Fifth Edition (DSM-V; CAPS-5)
- Ability to give signed, informed consent in English
- Normal or corrected-to-normal vision
- Participants who are on a stable dose of selective serotonin reuptake inhibitor (SSRI) antidepressants for 2 months (3 months if they are on sertraline), or who have been un-medicated for at least 2 months, will be allowed to participate in this study
- At the time of recruitment, patients must have no intention of changing their medication or psychotherapy during the 2.5-month period of the intervention
- Research group must be able to identify a trauma-related target region in or immediately adjacent to the amygdala

**Exclusion Criteria**

- Any primary psychiatric diagnosis of a current major mood disorder, psychotic disorder, autism, mental retardation, or DSM-5 substance use disorder of mild or greater severity (2 or more symptoms) in the past 30 days. Comorbid mood and anxiety disorders will be permitted if they are not the primary focus of clinical attention
- Any history of psychosis or mania
- Active suicidality within past year, or history of suicide attempt in past 2 years
- Any contraindication to MRI scanning (severe claustrophobia, ferromagnetic metal in body, etc.)
- Pregnancy
- Any unstable medical or neurological condition
- Any history of severe past drug dependence (i.e., a focus of clinical attention or a cause of substantial social or occupational difficulty)
- Any history of brain surgery, of penetrating, neurovascular, infectious, or other major brain injury, of epilepsy, or of other major neurological abnormality (including a history of traumatic brain injury [TBI] with loss of consciousness for more than 24 hours or posttraumatic amnesia for more than 7 days)
- Significant hearing loss or severe sensory impairment
- Any psychotropic medication other than a stable dose of selective serotonin reuptake inhibitors (SSRIs)
- Any change in accepted psychotropic medication within the past 2 months
- Active engagement in cognitive-behavioral therapy or any evidence-based PTSD psychotherapy (Cognitive Processing Therapy [CPT], Prolonged Exposure [PE], Eye Movement Desensitization and Reprocessing [EMDR]) initiated within the past 3 months; continuation of established maintenance supportive therapy will be permitted
- Enrollment in another research study testing an experimental/clinical/behavioral intervention intended to affect symptoms initiated within the last 2 months, or intended enrollment within the next 2.5 months

Other details of the current trial can be found on [clinicaltrials.gov/ct2/show/NCT03574974](https://clinicaltrials.gov/ct2/show/NCT03574974).

**Figure S1. CONSORT flow diagram**

**
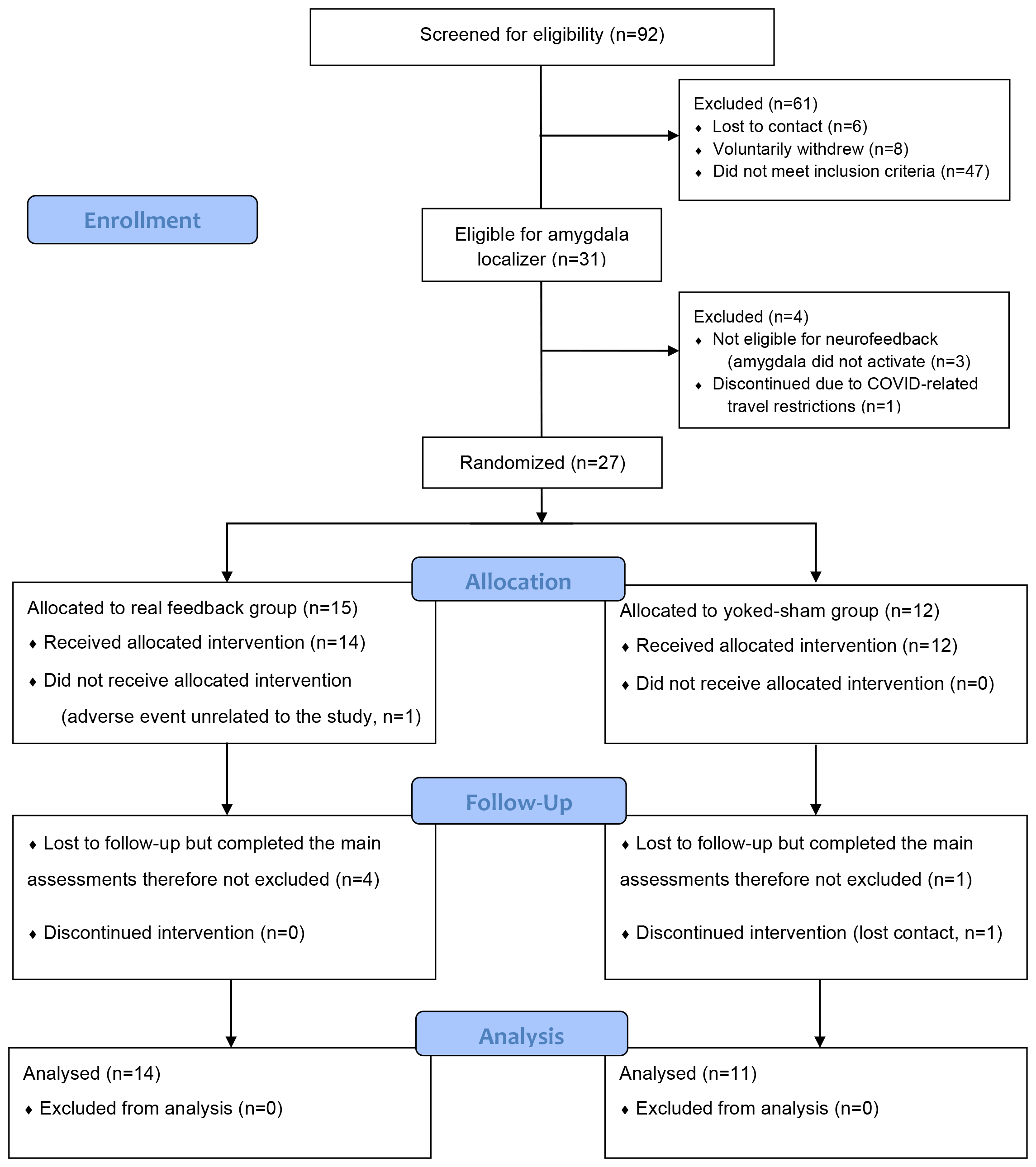
**

**Self-regulation strategies during neurofeedback training**

Before the first neurofeedback session, both groups of participants met with a clinical psychologist for a strategy development session. This clinical psychologist was blind to their intervention assignment. During this session, the participants were provided with a number of individualized mental strategies that might help them to decrease their amygdala activity and anxiety level after trauma recall during the forthcoming neurofeedback runs. Commonly discussed approaches included progressive relaxation [1], mindfulness [2], and reappraisal of emotion [3]. The goal of this procedure is to ensure participants in both groups have some cognitive strategies that may be helpful for controlling amygdala activity at the beginning of training.

During training sessions, the participants were instructed to judge the success of each cognitive strategy based on the feedback signal which allows them to discover and practice the most effective strategies for each individual.

**Imaging parameters and offline preprocessing**

MRI data were collected on a 3-Tesla MAGNETOM Prisma Fit system (Siemens Medical Solutions, Erlangen, Germany; version VE11B) using the 64-channel head coil. We used the same T2* weighted echo-planar imaging sequence (with various length) for all the functional scans: GRAPPA factor acceleration factor = 2, bandwidth = 2520 Hz/Px, TR = 2000ms, TE = 25ms, flip angle = 80 degrees, voxel size = 3.1×3.1×3.0 mm, FoV = 200mm^2^, forty-one interleaved AC-PC aligned slices per volume. To assist spatial registration to the standard space, high-resolution structural data were collected with an MPRage sequence: TR = 2530ms, TE = 2.81ms, flip angle = 7 degrees, 1mm^3^ isotropic voxel, FoV = 256mm^2^, 176 sagittal slices per volume.

During offline preprocessing, functional data from each task were aligned to the first volume using an in-house script based on SPM8 functions. A functional run was discarded if the average frame-wise displacement was higher than 0.25mm during the run.

The MPRage structural images were skull-stripped by the brain extraction tool in FSL (v6.0.1). These extracted brains were normalized to the MNI template using the non-linear registration tool in BioImage Suite (v3.5).

**Localizer task**

The participants underwent a localizer session to determine their eligibility for the study as well as to identify the amygdala region for the neurofeedback intervention. Each of the three runs lasted 5 minutes during which participants were exposed to the trauma audio clips with the highest rank. They were instructed to listen to the clips without controlling for their negative emotional responses. Similar to the control task, the rest (80s) and symptom provocation (60s) blocks were separated by 1-back blocks (40s) for a cleaner baseline (Figure S2).

**Figure S2. Illustration of a localizer run**


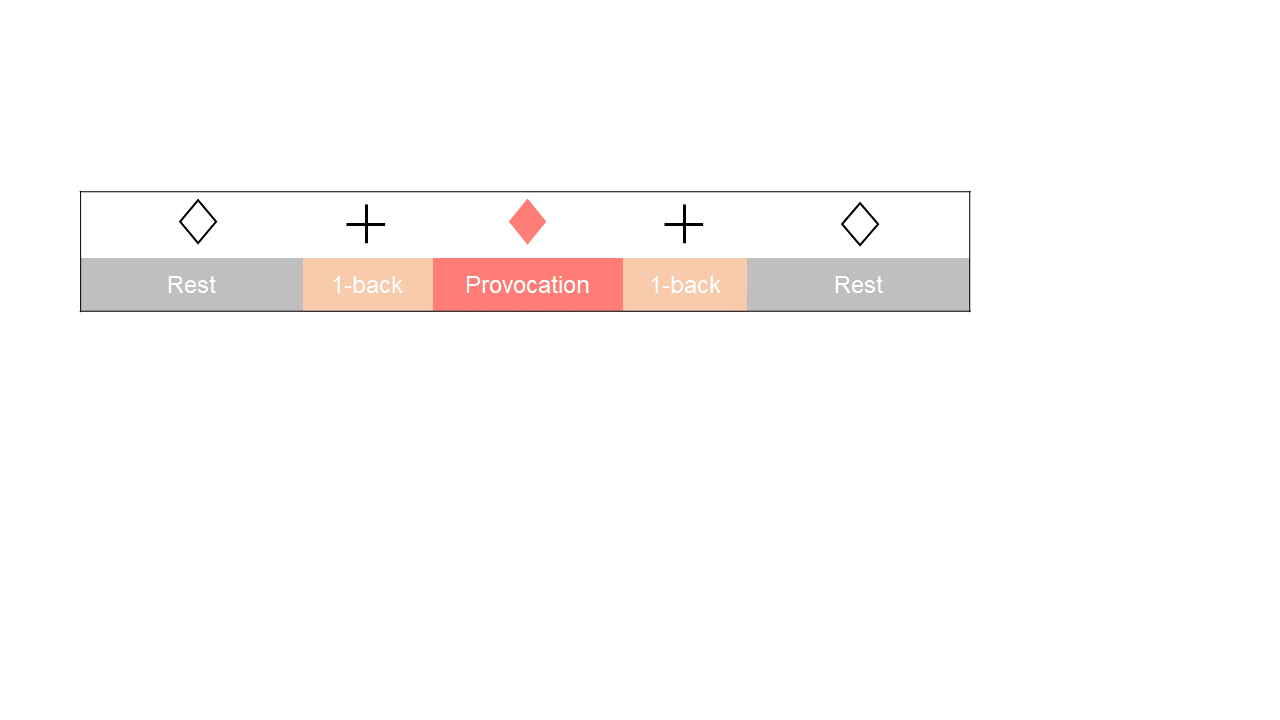


**GLM analyses**

Generalized Linear Model (GLM) analyses were performed in individual space. We used BioImage Suite to build GLMs for the localizer and control tasks with all but the rest condition included in the models as task regressors. The six rigid-body head movement parameters [4] and scanner drift (first-order) were added as nuisance regressors.

For the untrained control task runs, the downregulation beta maps were spatially smoothed using a 6mm FWHM kernel then registered to the MNI space using the transformation matrices generated from the structural registration step above after registering these beta maps to the structural images in FSL. Average beta values during downregulation were extracted from the target region of the amygdala (originally defined as the 30 most active voxels for each subject in the functional space of the localizer and then transformed to MNI space via the same registrations as the beta maps). This downregulation value averaged across two control runs (separately at screening, 30-day and 60-day follow-up) served as the measurement for amygdala control.

**Table S1. Baseline characteristics of the completers**

|  | **Active Group (*n =* 14)** | | **Control Group (*n =* 11)** | |
| --- | --- | --- | --- | --- |
|  | **Mean (μ)** | **Std dev (σ)** | **Mean (μ)** | **Std dev (σ)** |
| **Age, Years** | 40.2 | 14.27 | 50.36 | 12.78 |
| **CAPS-5** | 33.71 | 7.99 | 39.73 | 9.27 |
| **PCL-5 Rating** | 43.43 | 13.42 | 47.45 | 14.03 |
|  | **Count (n)** | **Percentage (%)** | **Count (n)** | **Percentage (%)** |
| **Male Gender** | 3 | 21.43 | 1 | 9.09 |
| **Race** |  |  |  |  |
| White | 14 | 100 | 11 | 100 |
| **Ethnicity** |  |  |  |  |
| Hispanic | 3 | 21.43 | 1 | 9.09 |
| **Medication Status** |  |  |  |  |
| No medication | 2 | 14.29 | 2 | 18.18 |
| Antidepressants | 7 | 50 | 2 | 18.18 |
| Anticonvulsant | 0 | 0 | 2 | 18.18 |
| Stimulants | 1 | 7.14 | 0 | 0 |
| Medical Marijuana/Cannabinoids (CBD Oil) | 1 | 7.14 | 3 | 27.27 |
| Unknown | 1 | 7.14 | 0 | 0 |
| **Additional Diagnoses** |  |  |  |  |
| MDD | 5 | 35.71 | 5 | 45.45 |
| MDD in partial remission | 2 | 14.29 | 2 | 18.18 |
| MDD in full remission | 5 | 35.71 | 2 | 18.18 |
| Dysthymic Disorder | 0 | 0 | 1 | 9.09 |
| OCD | 3 | 21.43 | 0 | 0 |
| Social Phobia | 5 | 35.71 | 3 | 27.27 |
| GAD | 2 | 14.29 | 2 | 18.18 |
| Body Dysmorphic Disorder | 2 | 14.29 | 2 | 18.18 |
| Panic Disorder with Agoraphobia | 1 | 7.14 | 1 | 9.09 |
| Panic Disorder without Agoraphobia | 1 | 7.14 | 3 | 27.27 |
| Agoraphobia without History of Panic Disorder | 1 | 7.14 | 0 | 0 |
| Specific Phobia (heights) | 0 | 0 | 1 | 9.09 |

CAPS, Clinician-Administered PTSD Scale; GAD, Generalized Anxiety Disorder; MDD, Major Depressive Disorder; OCD, Obsessive-Compulsive Disorder; PCL, PTSD Checklist

**Change in PCL-5 score**

PTSD Checklist for DSM-5 (PCL-5) was assessed during all visits. To compare with the CAPS-5 data we computed change from the individual baseline at the 30-day and 60-day follow-up time points and ran pooled t-tests on these change scores. Although the active group showed qualitatively greater reduction in the PCL score for both timepoints, the t-tests did not reveal a difference between the two intervention groups at either 30-day (p = 0.827) or 60-day (p = 0.476 ) follow-up for PCL-5. However, repeated-measures ANOVA does suggest a significant main effect of time (p < 0.001) without an interaction between time and intervention (p = 0.352).

**References**

1. Jacobs E. Progressive relaxation: physiological and clinical investigation of muscular states and their significance in psychology and medical practice. University of Chicago Press, Chicago1929.

2. Brown KW, Ryan RM. The benefits of being present: mindfulness and its role in psychological well-being. *Journal of personality and social psychology* 2003; **84**(4)**:** 822.

3. Buhle JT, Silvers JA, Wager TD, Lopez R, Onyemekwu C, Kober H *et al.* Cognitive reappraisal of emotion: a meta-analysis of human neuroimaging studies. *Cerebral cortex* 2014; **24**(11)**:** 2981-2990.

4. Friston KJ, Williams S, Howard R, Frackowiak RS, Turner R. Movement-related effects in fMRI time-series. *Magn Reson Med* 1996; **35**(3)**:** 346-355.
